# Toxoplasma encephalitis in HIV/AIDS patients admitted to the Douala general hospital between 2004 and 2009: a cross sectional study

**DOI:** 10.1186/1756-0500-6-146

**Published:** 2013-04-12

**Authors:** Henry Namme Luma, Benjamin Clet Nguenkam Tchaleu, Yacouba Njankouo Mapoure, Elvis Temfack, Marie Solange Doualla, Marie Patrice Halle, Henry Achu Joko, Sinata Koulla-Shiro

**Affiliations:** 1 Internal Medicine Unit, Douala General Hospital, P.O. Box 4856, Douala, Cameroon; 2 Faculty of Medicine and Biomedical Sciences, University of Yaoundé 1, Yaoundé, Cameroon; 3 Université des Montagnes, Bagangté, Cameroon; 4 Faculty of Medicine and Pharmaceutical Sciences, University of Douala, Douala, Cameroon

**Keywords:** Toxoplasma encephalitis, Case fatality, Altered sensorium, HIV-infected

## Abstract

**Background:**

It is estimated that about a third of the world’s population is chronically infected with *Toxoplasma gondii*. Toxoplasma encephalitis (TE), which occurs as a reactivation of quiescent chronic infection, is one of the leading causes of central nervous system (CNS) infection in AIDS. Its diagnosis in most centres still remains difficult. We opted to describe the clinical and radiological features of TE as well as in-hospital outcome and its associated factors.

**Methods:**

We carried out a cross sectional study on the clinical case notes of adult patients admitted and treated for TE at the Douala General Hospital, Cameroon between January 1^st^ 2004 to December 31^st^ 2009.

**Results:**

Of 672 patients admitted during the study period, 14.4% (97/672) had TE. The mean age was 36.9 ± 14.1 years and the median CD4 cell count was 68/mm^3^ (IQR): 43 – 103). Headache and fever were the most common presenting symptoms in 92.8% (90/97) and 87.6% (85/97) of patients. Annular contrast enhanced lesions were the most common brain scan finding in 81.4% (79/97) of patients. In-hospital mortality was 29.9% (29/97). Altered sensorium, presence of focal signs, neck stiffness and low CD4 cell count were factors associated with mortality. Adjusting for low CD4 count, altered sensorium remained strongly associated with fatality, adjusted odd ratio (AOR) 3.5 (95% CI 1.2 – 10.5).

**Conclusion:**

Toxoplasma encephalitis is common among AIDS patients in Douala. Its high case fatality warrants adequate and compliant prophylactic therapy in severely immune depressed patients as well as early initiation of antiretroviral therapy in HIV-infected patients.

## Background

Globally, about a third of the world’s population is chronically infected with *Toxoplasma gondii*[1]. Toxoplasma encephalitis (TE) is the most frequent cause of focal central nervous system (CNS) infection complicating Acquired Immune Deficiency Syndrome (AIDS) [2]. It has been shown to occur by reactivation of quiescent infection [3,4] as a result of loss of cellular immunity. In the CNS, the predominant neuropathologic feature of TE is multifocal necrotizing encephalitis [5] which progresses to parenchymal abscesses with necrosis and surrounding inflammation [6]. This life threatening infection increases in frequency with severity of immune depression [2,7] and has a variable worldwide seroprevalence [8]. In some high income settings with high seroprevalence, it has been estimated that in the absence of prophylaxis 30 to 40% of patients with AIDS will develop TE [9]. Like most CNS diseases in AIDS, diagnosis of TE is often difficult such that in clinical practice treatment of TE is usually initiated upon presumption based on clinical and radiological features as well as response to treatment [2,10]. The advent of highly active antiretroviral therapy (HAART) and its effectiveness in improving the immunological status of AIDS patients has been accompanied by a decrease in incidence of TE in high income settings [11]. In sub Saharan Africa where about two thirds of the global HIV burden is found [12] the picture might be different because many patients still present with severe immune depression and HAART coverage is not yet universal. More so, access to health care is limited, a situation which might underestimate the burden of TE in AIDS. This was the impetus behind this hospital based study whose aim was to define the clinical and radiological features of patients with TE, determine in-hospital outcome and its associated factors.

## Methods

### Study setting

The study was carried out at the Douala General Hospital (DGH), a tertiary referral hospital in Douala, the largest city in Cameroon. It has a capacity of 320 beds and is the most specialised in the sub region. Prior to the study, local institutional ethical clearance of the Douala General Hospital was sought and obtained. At the DGH, HIV diagnosis is made according to guidelines of the Cameroon National AIDS control programme [13] by antibodies detection on two successive samples using a third generation ELISA test BIOREX® (Biorex Diagnostics Limited, Antrim, United Kingdom). If both are positive, a third sample is collected and tested using Genie® III HIV-1/HIV-2 Assay (Bio-Rad Diagnostics, France) to specify whether HIV 1 or HIV 2. These three tests if positive, the patient is declared positive for HIV. In case of any discordance, testing is done with Western blot (New LAV blot, Diagnostics, Pasteur). In this institution, diagnosis of CNS disease follows an algorithm that uses clinical, biological and radiological features as well as patient response to treatment. When a patient presents with clinical signs and/or symptoms relevant to CNS disease, firstly a computerised tomographic (CT) brain scan is done in search of space occupying lesions and/or signs of raised intracranial pressure (IP). In the absence of space occupying lesions, raised IP or any contraindication, a lumbar tap is then done for cerebrospinal fluid analysis.

### Study population and diagnosis of TE

We carried out a cross sectional analysis of the files of all adult (>18 years) HIV-1 patients admitted in the internal medicine unit of the DGH and treated for TE during the period spanning January 1^st^ 2004 and December 31^st^ 2009. The diagnosis of TE was presumed when a patient presented with symptoms (headache, seizures, fever, and altered sensorium) and/or signs (neck stiffness, focal signs) together with single or multiple ring enhanced lesions on CT scan imaging. The diagnosis was only confirmed when a patient improved on anti-toxoplasma treatment which is mainly cotrimoxazole in our centre. From the files of these patients, we collected socio-demographic, clinical, radiological information relevant to our study on a case reporting form (CRF).

### Data analysis

The data from the CRF was entered into Epidata 3.1 and exported to STATA 11.2 (Stata Corporation, College Station, Texas) for analysis. Clinical and radiological features were categorised as either present or absent, expressed as a percentage of the study population and reported with their 95% confidence interval (95% CI). The main outcome of interest was in-hospital death which was expressed as a percentage of the study population to obtain case fatality rates, it was then categorised as present or absent and through Mantel Haenszel method measure of association with covariates was done. Odd ratios (OR) with their 95% CI were presented. A threshold of association was defined for a two sided p value of less than 0.05.

## Results

### Characteristics of study population

During the study period, 672 HIV infected patients were admitted to the Internal Medicine unit of who 97 fulfilled eligibility for the study, giving a prevalence of toxoplasma encephalitis of 14.4% (97/672). Of the 97, 52.6% (51/97) were females (Table 1). The mean age of the study population was 36.9 ± 14.1 years and median CD4 cell count was 68/mm^3^ [Interquartile range (IQR): 43 – 103)], with 73.2% (71/97) having less than 100 cells/mm^3^. Men had lower mean CD4 cell counts than women (p < 0.001).

**Table 1:** General characteristics of 97 HIV infected patients with presumed toxoplasma encephalitis

**Age groups**	**N (%)**
<30	37 (38.1)
30 – 39	26 (26.8)
40 – 49	17 (17.5)
50 – 59	11 (11.3)
>60	6 (6.2)
Sex	
Male	46 (47.4)
Female	51 (52.6)
Marital status	
Single	27 (27.8)
Married	69 (71.1)
Divorced	1 (1.0)
CD4 cell count per mm^3^	
<50	29 (29.9)
50 – 99	42 (43.3)
>100	26 (26.8)

### Clinical and radiological features of patients with TE

Fever and headache were the most common symptoms (Table 2). Neck stiffness was found in 15.5% of patients and these patients had the lowest median CD4 cell count (Table 2). On CT scan, patients who presented with nodular enhanced lesions also had the lowest median CD4 cell count (Table 2).

**Table 2:** Clinical presentation, head CT scan findings and median CD4 counts of 97 HIV positive patients with presumed toxoplasma encephalitis

	**N**	**Prevalence**	**95% Confidence Interval (CI)**	**Median CD4 count (IQR)**
Symptoms				
Headache	90	92.8	87.5 – 98.0	67 (41 – 92)
Fever	85	87.6	80.9 – 94.3	61 (41 – 90)
Seizure	56	57.7	47.7 – 67.7	47 (28 – 71)
Altered sensorium	23	23.7	15.1 – 32.3	29 (17 – 41)
Clinical signs				
Focal signs	64	65.9	56.4 – 75.6	53 (32 – 73)
Neck stiffness	15	15.5	8.1 – 22.8	33 (27 – 43)
CT Scan findings				
Annular enhancement	79	81.4	73.6 – 89.3	58 (36 – 87)
Nodular enhancement	3	3.1	0.4 – 6.6	33 (12 – 45)
Pre-suppurative encephalitis	15	15.5	8.1 – 24.0	60 (41 – 89)

### In-hospital outcome of patients with TE and its associated factors

A total of 29 patients died giving a case fatality rate of 29.9% (Table 3). Their median CD4 cell count was 41/mm^3^ (IQR: 24 – 75). Men were more likely to die than women though this was not statistically significant (Table 3). The presence of altered sensorium, focal signs, neck stiffness and low CD4 cell count were all predictors of mortality (Table 3). After adjusting for low CD4 count, altered sensorium remained strongly associated with fatality, adjusted odd ratio (AOR) 3.5 (95% CI 1.2 – 10.5, p = 0.02).

**Table 3:** Case fatality rate of presumed toxoplasma encephalitis and associated factors

	**N**	**Number of death**	**% Death of study population**	**OR (95% CI)**	**P value**
Age					
<40	63	22	22.7	1	0.1
>40	34	7	7.2	0.5 (0.2 – 1.3)	
Sex					
Female	51	11	11.8	1	0.06
Male	46	18	18.6	2.3 (0.9 – 5.8)	
Altered sensorium					
No	74	16	16.5	1	0.002
Yes	23	13	13.4	4.7 (1.6 – 13.5)	
Focal signs					
No	33	4	4.1	1	0.006
Yes	64	25	25.8	4.6 (1.4 – 15.6)	
Seizures					
No	41	7	7.2	1	0.02
Yes	56	22	22.7	3.1 (1.1 – 8.6)	
Neck stiffness					
No	82	21	21.6	1	0.03
Yes	15	8	8.2	3.3 (1.1 – 10.2)	
CD4 cell count					
>100/mm^3^	26	3	3.1	1	0.001
<100/mm^3^	71	26	26.8	4.4 (1.5 – 16.9)	
**Total**	**97**	**29**	**29.9**	**/**	**/**

## Discussion

Our study showed that the prevalence of TE among HIV infected patients was 14.4%, a finding similar to that of another study [14]. It has been shown that in situations of severe immune deficiency the prevalence of TE reflects that of latent toxoplasma infection [15]. Seroprevalence of anti-toxoplasma antibodies which is a proxy of the burden of latent infection could predict the burden of TE especially in areas with high HIV prevalence. In one recent study in Cameroon, the seroprevalence of toxoplasma antibodies in HIV/AIDS patients was a high 69.9% [16]. In this setting where HAART coverage is low [17] and HIV patients still present with severe immune deficiency, TE might be a major cause of morbidity and mortality whose real picture is still unclear. This therefore calls for systematic primary anti-toxoplasma prophylaxis for patients with low CD4 counts.

Clinically, patients with TE usually have a sub-acute presentation over several weeks with symptoms and signs usually limited to the CNS including headaches, fever, psychomotor or behavioural abnormalities, confusion, focal signs and seizures [18]. In our study, headaches and fever were the most common symptoms. Other studies similarly found headaches and fever present in over 50% of patients [14,19]. The prevalence of seizures in our study was similar to that of a Malian study [20] but higher than that in another town in Cameroon [21]. This difference might be because of the referral nature of our institution whereby seizures being a dramatic clinical presentation, patients are more easily referred. The occurrence of focal signs in our study was concordant with that of other studies [20-22]. In view of the above findings, CNS symptoms and signs in an HIV infected patient should be promptly investigated and early empirical treatment for possible TE commenced [23].

Brain CT scan examination has been shown to be the most useful means by which the diagnosis of TE could be presumed [14]. All our patients had a brain scan; this is not always the case in most hospitals in Cameroon due to lack of CT scan machines. In such cases, empirical treatment is advised even though it might lead to multiple drug administration targeting numerous differential diagnoses especially as the clinical picture of TE varies and is not specific. This approach however could help reduce TE –associated mortality. On the other hand, CT scan findings are not pathognomonic: differential diagnoses include tuberculoma and primary CNS lymphoma depending on the setting [23]. The typically described ring enhancing lesion was found in 81.4% of our patients, similar to another study [22].

TE is more common in the advanced stage of HIV disease when CD4 count is low [2,7]. The risk of opportunistic infections increases markedly when values fall below 200/mm^3^[15]. Many studies have reported a significant relationship between CD4 cell counts of less than 100 cells/mm^3^ and the development of TE [2,7]: 73.2% of our patients had CD4 counts of less 100/mm^3^.

The case fatality was 29.9% in our study. Though similar to that in other studies [14,22] such high case fatality was surprising because the disease can be prevented by adequate prophylactic therapy and more so treatment is readily available [24]. In our setting, this high case fatality could be explained by the fact that on the one hand, most physicians neglect primary prevention of TE and on the other, patients on prophylaxis may not be compliant with their treatment. Furthermore, empirical treatment even when indicated is usually commenced late because many patients seek medical care only when their clinical state markedly deteriorates. This therefore warrants continued awareness by health care providers on primary prevention and early treatments as this could considerably reduce mortality. Low CD4 count was a predictor of mortality and irrespective of CD4 count, altered sensorium, an index of severe CNS disease was independently associated with mortality. This is a reflection of the health system of our setting where patients only seek care when their disease is at the terminal stage. Patients’ sensitisation on the importance of early medical care as well as the reorganisation of the health system to ease access to care, are important steps to reduce TE-associated mortality.

There were some limitations in our study. The diagnosis of TE which remains presumptive may have led to under estimation or over estimation of the burden of TE. Response to treatment which was used as an index to confirm diagnosis of TE was useful only for patients with good response suggesting that some deaths might have been due to a cause other than TE. Moreover, information on anti-toxoplasma prophylaxis and HAART were not included in our analysis because they were consistently not available in patient records.

## Conclusion

TE is a common cause of morbidity and mortality among severely immune depressed HIV-infected patients in Cameroon. Though relatively easy to treat, a definitive diagnosis of TE is still difficult in most centres in Cameroon. The case fatality rate of TE is high; hence primary prophylaxis with adequate compliance should be instituted among patients with severe immune depression as well as early initiation of antiretroviral therapy in HIV-infected patients to avoid severe immune depression.

## Competing interest

The authors declare no conflict of interest.

## Authors’ contributions

HNL, BCNT, YNM, ET and SKS designed the study. BCNT, YNM and HAJ collected the data. HNL and ET analysed the data and did manuscript write up, assisted by MPH and MSD. SKS, MSD and HNL proofread the manuscript. All authors agreed with the final manuscript to be submitted.
